# Comparative analysis of batch correction methods for FDG PET/CT using metabolic radiogenomic data of lung cancer patients

**DOI:** 10.1038/s41598-023-45296-9

**Published:** 2023-10-25

**Authors:** Hyunjong Lee, Sujin Seo, Sungho Won, Woong-Yang Park, Joon Young Choi, Kyung-Han Lee, Se-Hoon Lee, Seung Hwan Moon

**Affiliations:** 1 Department of Nuclear Medicine, Samsung Medical Center, Sungkyunkwan University School of Medicine, 81 Irwon-ro, Gangnam-gu, Seoul, 06351 Republic of Korea; 2 Department of Public Health Science, Graduate School of Public Health, Seoul National University, Gwanak_1 Gwanak-ro, Gwanak-gu, Seoul, 08826 Republic of Korea; 3 Department of Molecular Cell Biology, Samsung Medical Center, Samsung Genome Institute, Samsung Advanced Institute of Health Science and Technology, Sungkyunkwan University School of Medicine, Seoul, Republic of Korea; 4 Division of Hematology/Oncology, Department of Medicine, Samsung Medical Center, Sungkyunkwan University School of Medicine, Seoul, Republic of Korea

**Keywords:** Cancer imaging, Genetics research

## Abstract

In radiomics research, the issue of different instruments being used is significant. In this study, we compared three correction methods to reduce the batch effects in radiogenomic data from fluorodeoxyglucose (FDG) PET/CT images of lung cancer patients. Texture features of the FDG PET/CT images and genomic data were retrospectively obtained. The features were corrected with different methods: phantom correction, ComBat method, and Limma method. Batch effects were estimated using three analytic tools: principal component analysis (PCA), the *k*-nearest neighbor batch effect test (kBET), and the silhouette score. Finally, the associations of features and gene mutations were compared between each correction method. Although the kBET rejection rate and silhouette score were lower in the phantom-corrected data than in the uncorrected data, a PCA plot showed a similar variance. ComBat and Limma methods provided correction with low batch effects, and there was no significant difference in the results of the two methods. In ComBat- and Limma-corrected data, more texture features exhibited a significant association with the TP53 mutation than in those in the phantom-corrected data. This study suggests that correction with ComBat or Limma methods can be more effective or equally as effective as the phantom method in reducing batch effects.

## Introduction

F-18 fluorodeoxyglucose positron emission tomography/computed tomography (FDG PET/CT) is a nuclear imaging modality based on the mechanism of greater activation of glucose metabolism in malignant tissue than in normal tissue^1,2^. The standardized uptake value (SUV) is a quantitative value provided by FDG PET/CT that represents the glucose uptake of tissue. The maximum SUV (SUVmax) is the most representative value and is known to have powerful diagnostic and prognostic significance in various malignancies. Despite the usefulness of SUVmax, it has the limitation of being able to demonstrate only a specific voxel value in a volume-of-interest (VOI). Even volumetric parameters, such as metabolic tumor volume (MTV) and total lesion glycolysis (TLG), are inappropriate for evaluating the overall metabolic pattern of tumor tissue.

Therefore, many approaches have been employed to assess the metabolic pattern of tumor tissue using radiomics analysis in FDG PET/CT images. Radiomics is a method that extracts varying features from medical images using diverse algorithms^3^. Many metabolic texture parameters have been used to demonstrate a significant association with tumor biology or the prognosis of cancer patients^4,5^. However, the use of different instruments in radiomics research remains a significant issue. In the clinical field, many multi-center trials with different PET/CT scanners are actively conducted. Owing to differences in image acquisition protocols, voxel sizes, and reconstruction parameters, correction of metabolic texture parameters from different instruments are required for pre-processing before radiomics analysis is conducted. Phantom correction is a conventional correction method that unifies parameters from different instruments based on the value ratio from each instrument. Harmonization is a statistical method that corrects the batch effects of different instruments^6^. There have been several studies to evaluate the effect of the harmonization method to reduce the batch effects of image parameters^7–9^. However, no study has been conducted to compare the effects of different correction methods in terms of their association with the genetic characteristics of lung cancer.

In this study, we compared three correction methods to reduce the batch effects of radiomic data from FDG PET/CT images of lung cancer patients. Texture features were corrected using different correction methods: phantom correction, the ComBat method in the ‘sva’ package, and the ‘removeBatchEffect’ function in the ‘Limma’ package in R software, respectively. The batch effects of the corrected features were compared between each method. In addition, the associations of corrected features and gene mutations were also compared.

## Methods

### Subjects

**Figure 1:**
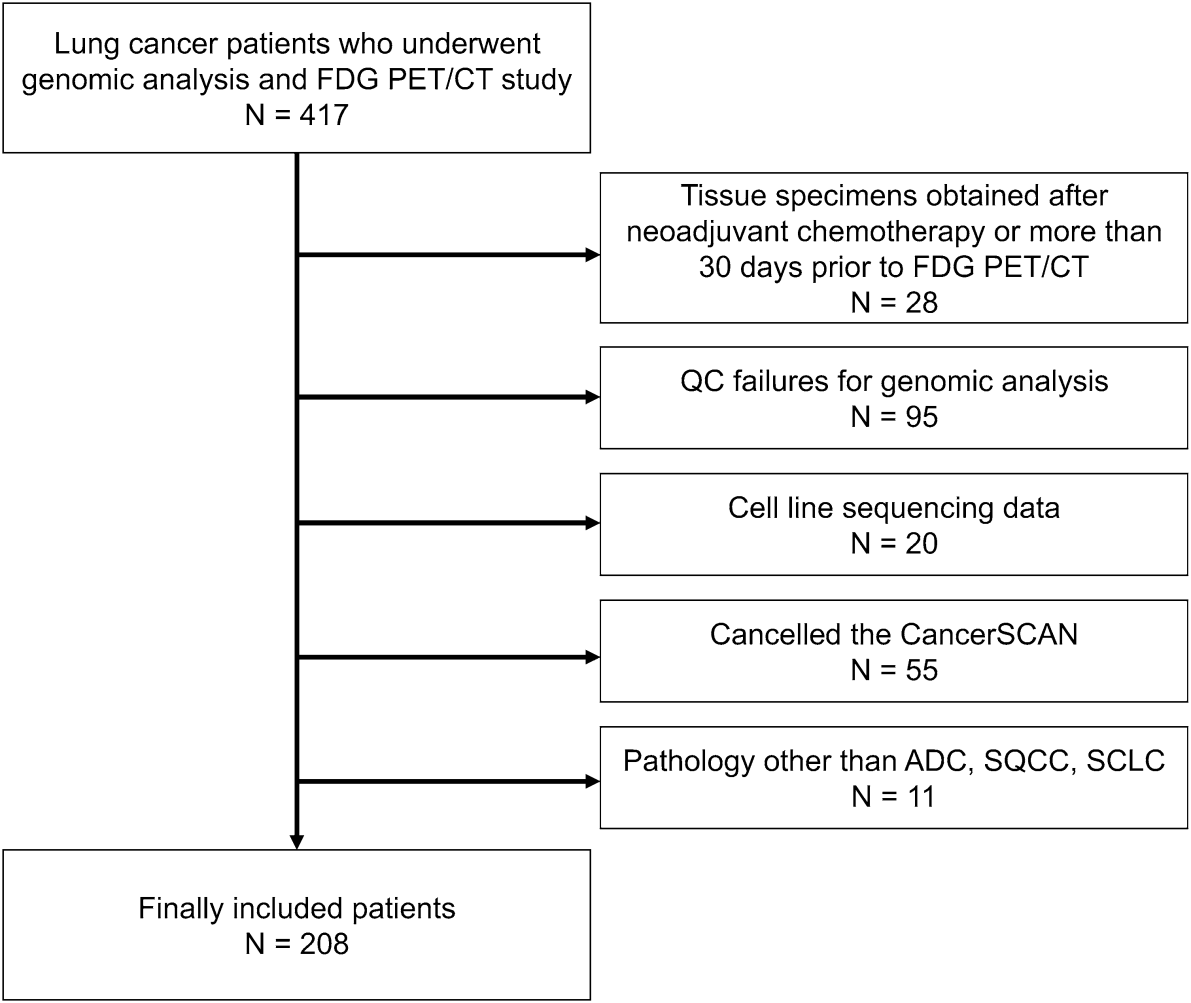
Flowchart of the study design.

In this study, we enrolled subjects from the same candidates as in a previous study^10^. The study candidates were 417 patients with histologically confirmed lung cancer who were enrolled in a database at the Samsung Genome Institute and who underwent FDG PET/CT. Genetic profiles of their tumor tissues were created using the CancerSCAN, next-generation sequencing (NGS)-based targeted-sequencing platform designed at our institution. All patients agreed that their data could be used in other studies. Within that candidate pool, we excluded 28 patients whose tumor tissues were obtained for genomic analysis after neoadjuvant therapy more than 30 days prior to their PET/CT. Of the remaining 389 patients, we excluded 95 patients whose CancerSCAN results failed quality control, 20 patients with cell line sequencing data, 55 patients who cancelled their CancerSCAN, and 11 patients with cancers other than adenocarcinoma (ADC), squamous cell carcinoma (SQCC), or small cell lung cancer (SCLC). All PET scans in these patients were performed before treatment. Therefore, a total of 208 patients were finally included (Fig. 1). Samsung Medical Center institutional review board approved this retrospective cohort study. Due to the retrospective nature of the study, Samsung Medical Center institutional review board waived the need of obtaining informed consent. All methods were carried out in accordance with relevant guidelines and regulations.

### CancerSCAN

CancerSCAN is an NGS-based targeted-sequencing platform designed at our institution. The reliability of this assay was proved by robust analytic validation in previous studies, where the details of the experimental procedures were described^11–13^. CancerSCAN version 1 targets 83 genes, and version 2 targets 381 genes. The selected target genes for this customized platform were curated at the request of researchers and clinicians. These target genes were associated in the literature and public databases with targeted cancer therapies or therapy responses. Single nucleotide variants, small insertions/deletions, copy number variations, and gene fusions were detected using both existing and new algorithms. The variant calls were classified into four categories to reflect the mode and functional effect of the mutations and then condensed at the gene level. The four categories included (1) MUT: miss-sense mutation, (2) LoF: loss of function variant, including frame-shift insertion/deletion and stop-gain mutation, (3) CNV: copy number variation, and (4) FUSION: known driver gene fusion event.

### FDG PET/CT acquisition

All patients fasted for at least six hours and had blood glucose levels of less than 200 mg/dL at the time of their FDG PET/CT scans. Whole-body PET and CT images from the basal skull to mid-thigh were acquired 60 min after the injection of 5.0 MBq/kg FDG without intravenous or oral contrast on a Discovery LS or a Discovery STE PET/CT scanner (GE Healthcare, Milwaukee, WI, USA). Continuous spiral CT was performed with an 8-slice helical CT (140 keV, 40–120 mA; Discovery LS) or a 16-slice helical CT (140 keV, 30–170 mA; Discovery STE). An emission scan was then obtained from head to thigh for 4 min per frame in 2-dimensional mode (Discovery LS) or 2.5 min per frame in 3-dimensional mode (Discovery STE). PET images were reconstructed using a CT for attenuation correction by the ordered-subsets expectation maximization algorithm with 28 subsets and 2 iterations (matrix 128 × 128, voxel size 4.3 × 4.3 × 3.9 mm; Discovery LS) or the ordered-subsets expectation maximization algorithm with 20 subsets and 2 iterations (matrix 128 × 128, voxel size 3.9 × 3.9 × 3.3 mm; Discovery STE). Hereafter, the data from Discovery STE are denoted as ‘batch 1’ and those from Discovery LS are ‘batch 2.’

### FDG PET/CT image analysis

Image feature extraction was based on a previous study and used the gradient-based segmentation method (‘PET Edge’) in MIM version 6.4 software (MIM Software Inc., Cleveland, OH, USA). The target tumor was identified by an experienced nuclear medicine physician (S.H.M.) who was unaware of all clinical information except the target tumor site. As the physician dragged the cursor from the center of the target tumor to a point near the edge of the lesion, six axes interactively extended. The length of each axis was restricted when a large gradient was detected along that axis. Then, the software automatically outlined a three-dimensional VOI on the tumor. After performing gradient-based segmentation of the target tumor lesion, we extracted PET image features using the Chang-Gung Image Texture Analysis toolbox (CGITA, https://code.google.com/p/cgita), an open-source software package implemented in MATLAB (version 2012a; MathWorks Inc., Natick, MA, USA). A total of 86 PET features available in CGITA were measured on each segment, and all the features were included for analysis.

### Phantom correction

For phantom correction, FDG PET/CT images of a cylinder phantom (NEMA NU2-1994) were acquired by each instrument, Discovery LS and Discovery STE. Air, Teflon, and a hot cylinder were inserted. A hot cylinder phantom was filled by FDG solution with a concentration of 5 MBq/kg. The ratio of the hot cylinder to the background was set as 4:1. The texture parameters were obtained by the same method described above. Briefly, a VOI was drawn by the ‘PET Edge’ method on a hot cylinder, which was identified by an experienced nuclear medicine physician. The parameters of a drawn VOI were calculated by CGITA software. The ratio of each texture feature between the two instruments was calculated. The texture parameters of FDG PET/CT acquired from Discovery STE were corrected by the ratios calculated above.

### Harmonization

As statistical batch-effect adjustment methods, we considered the ‘removeBatchEffect’ function in the Limma R package^14^ and ComBat^15^, which were originally developed for expressions of RNA sequencing or microarrays, although they can be used for other data types. ComBat can make the mean and variance of the samples equal to either the global mean and variance, or those for the specified reference batch. Hereafter, the former is termed ‘ComBat (Global),’ while those for the given batch are referred to as ‘ComBat (Ref 1) or ‘ComBat (Ref 2)’. ‘removeBatchEffect’ in Limma R package employs a robust approach by incorporating batch information as a covariate within a linear modeling framework. It operates under the assumption that batch effects can be represented as linear additive effects. To address this, Limma fits a linear model to the data, including batch as a covariate. It then effectively mitigates batch effects by subtracting the estimated batch effect.

Finally, we assessed the existence of the batch effect by principal component analysis (PCA) plots, the rejection rates of the k-nearest neighbor batch effect test (kBET) method^16^, and silhouette scores^17^. If the principal component scores were clustered by batch, it may have suggested that the data were systemically inconsistent owing to the different batches. For subject $$i$$, let $$a_{i} $$ be the average distance to the other samples within the same batch and let $$b_{i}$$ be the minimum of the averaged distance from the samples in other batches. Then, the silhouette score, $$s_{i}$$, is defined by

$$ {\text{s}}_{{\text{i}}} = \frac{{b_{i} - a_{i} }}{{{\text{max}}\left( {a_{i} , b_{i} } \right)}} $$

where the larger value indicates the separation of batches, which leads to the batch effect. The kBET rejection rate considered the batch distribution of each sample’s neighborhood, and it tested whether the proportion of the batch of *k*-nearest neighbors of randomly selected samples was similar to the global batch proportion using Pearson’s $\chi^{2}$ test. The number of nearest neighbors, *k,* was set to $k = 10$, and the process was repeated 1000 times to calculate the rejection rate. A higher rejection rate meant that the proportions of local and global batches were significantly different. The means of the kBET rejection rate and silhouette scores with and without batch effect adjustment were compared with t-test method.

### Associative test between corrected features and gene mutations

The statistical association between each image feature and gene mutation was tested. For each gene, we checked the mutation, and the mutated gene was coded as ‘1’. Otherwise, it was coded as ‘0.’ In order to provide sufficient statistical power in the study, if the minor mutation frequencies were less than 0.05, they were removed, and the remaining 50 genes were tested by using logistic regression. For each gene mutation (dependent variable) and each image feature (independent variable), a separate logistic regression model was built to analyze the association between the presence of a gene mutation and the value of an image feature. The significance level was set to 0.05 and the multiple testing problem was adjusted with the Benjamini–Hochberg method. All statistical analyses were performed using R software (v. 4.0.4, R Foundation for Statistical Computing, Vienna, Austria).

## Results

### Harmonization of FDG PET/CT image features

**Figure 2:**
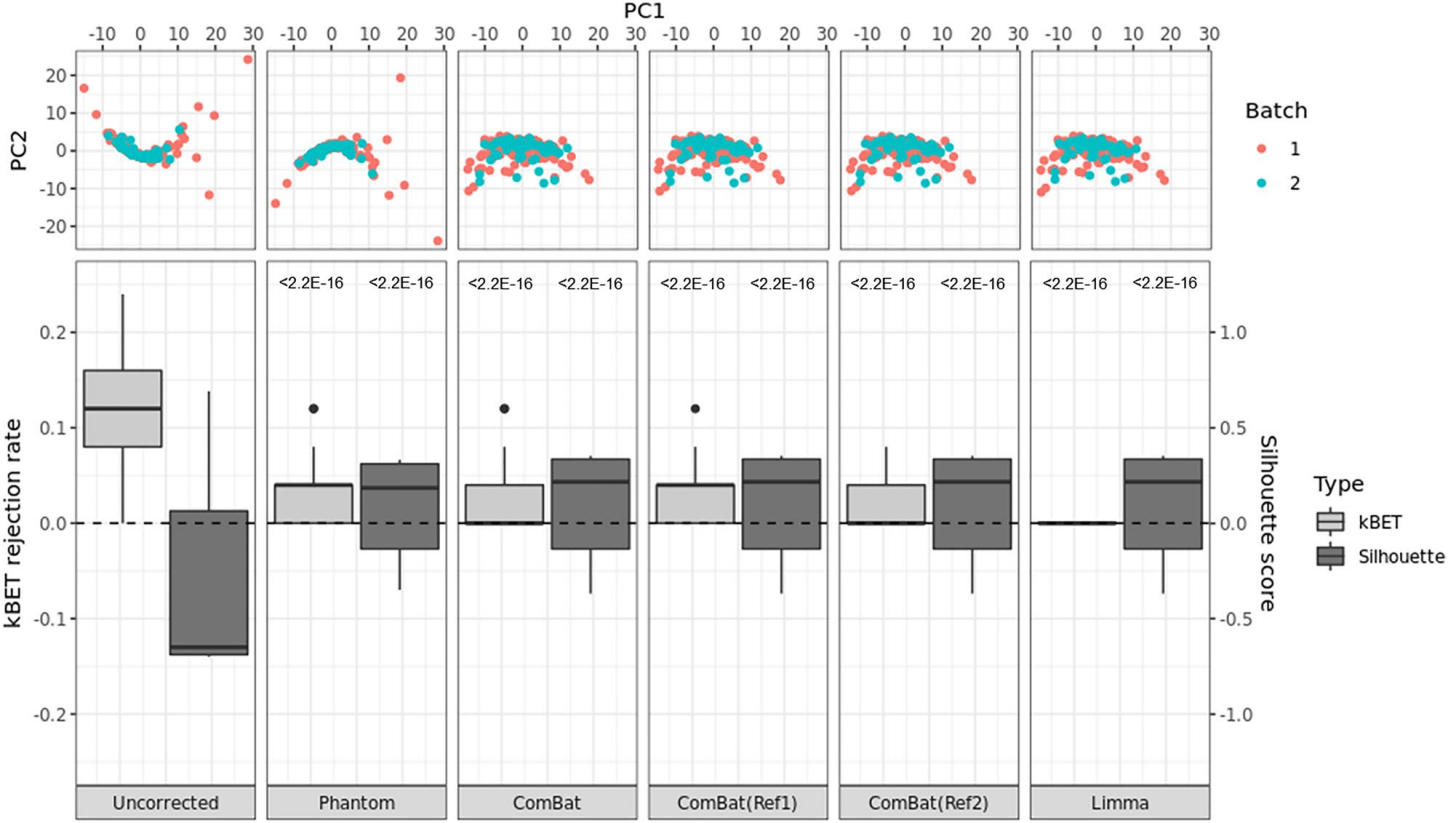
PCA plots (top) and box plots of kBET rejection rates and silhouette scores (bottom) by each batch correction method. The *p*-values from the t-test compared with uncorrected data are noted in the top of each box plot.

To identify the presence of the batch effect, the kBET rejection rate and silhouette score were calculated for image features before and after harmonization and conducting PCA. The PCA plots enabled evaluation of the distribution of data using a visual assessment. If the kBET rejection rate and silhouette score—parameters for measuring the batch effects—were closer to zero, it meant that there was a smaller batch effect between datasets. In Fig. 2, the PCA plots show substantial differences between batch 1 and batch 2 in uncorrected data and phantom-corrected data. For the uncorrected data and phantom-corrected data, subjects belonging to batch 1 data demonstrate greater dispersion with more outlying subjects. However, for ComBat- and Limma-corrected data, there are no significant differences between batch 1 and batch 2 (Fig. 2 upper panel). It is also shown that all harmonization methods lowered the kBET rejection rate and increased the absolute silhouette scores closer to zero (Fig. 2 lower panel). All *p*-values from the t-test comparing the results from the uncorrected data and corrected data are less than 0.05, which implies that the rejection rates and silhouette scores were significantly changed.

### Association between corrected features and gene mutations

**Figure 3:**
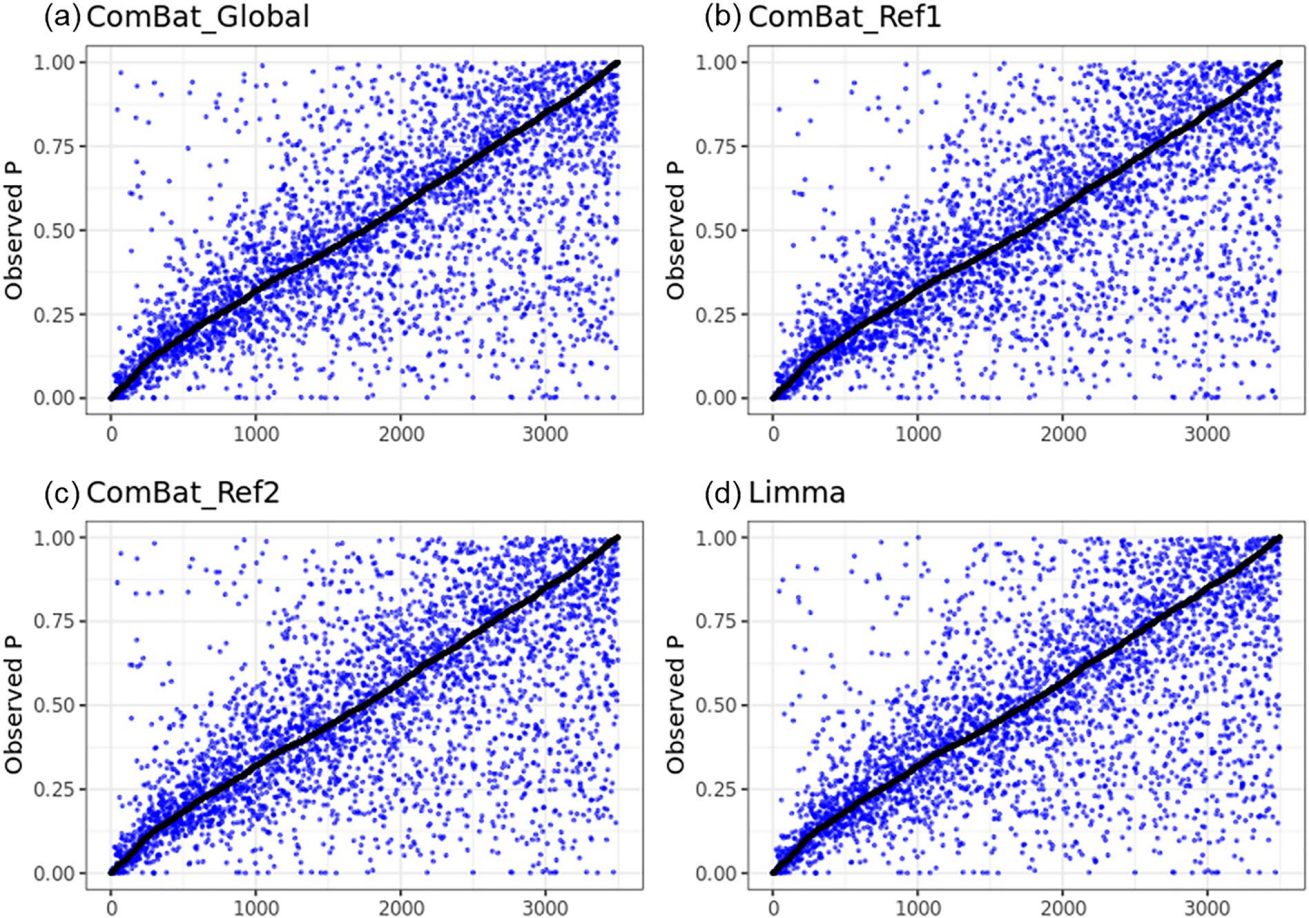
Plots of *p*-values of logistic regression for (**a**) ComBat (Global), (**b**) ComBat (Reference 1), (**c**) ComBat (Reference 2), and (**d**) Limma versus *p*-values for the phantom method (black dots in each box plot.

The *p*-values of logistic regression between the texture features and gene mutation profiles were plotted after adjusting the batch effects with ComBat (Global), ComBat (Ref 1), ComBat (Ref 2), Limma, and phantom methods (Fig. 3). The *p*-values from the phantom methods were compared with those from the other methods. The largest r^2^ was from Combat (Global), and the smallest r^2^ was from Limma (r^2^ = 0.4528 for Combat, r^2^ = 0.4325 for Limma). All significantly associated genes and image features in the phantom-corrected data were also significant in the data corrected by ComBat (Global). However, the reverse was not satisfied. There were two gene mutations demonstrating significant association with texture features, TP53 and IRS2. Three texture features—neighborhood intensity-difference coarseness, normalized co-occurrence entropy, and SUV statistics entropy—showed significant association with the TP53 mutation for all correction methods. Four texture parameters, co-occurrence contrast, co-occurrence dissimilarity, size-zone variability, and intensity variability showed significant association with the TP53 mutation only for ComBat and Limma methods in contrast to the phantom method (Table 1). The statistics are detailed in Table 2.

**Table 1:** Significant genes for each image feature after logistic regression.

Feature parent	Feature	Gene	Phantom	ComBat (Global)	ComBat (Ref 1)	ComBat (Ref 2)	Limma
Co-occurrence	Contrast	TP53	1	0.0081*	0.0087*	0.0095*	0.0088*
Co-occurrence	Dissimilarity	TP53	1	0.0286*	0.0309*	0.0322*	0.0300*
Intensity size zone	Size-zone variability	TP53	1	0.0087*	0.0096*	0.0099*	0.0088*
Neighborhood intensity difference	Coarseness	TP53	0.0121*	0.0126*	0.0133*	0.0107*	0.0368*
Neighborhood intensity difference	Strength	TP53	0.0099*	0.0301*	0.0323*	0.0290*	0.0968
Neighboring gray level dependence	Number nonuniformity	TP53	1	0.0486*	0.0524	0.0545	0.0536
Normalized co-occurrence	Entropy	TP53	0.0039*	0.0018*	0.0020*	0.0020*	0.0015*
SUV statistics	Entropy	TP53	0.0145*	0.0084*	0.0071*	0.0069*	0.0060*
SUV statistics	Mean SUV	IRS2	0.0043*	0.0465*	0.0445*	0.4419	0.1026
SUV statistics	SUV SD	TP53	1	0.0471*	0.0283*	0.0554	0.1270
Voxel alignment	Intensity variability	TP53	1	0.0268*	0.0287*	0.0302*	0.0299*

Benjamini–Hochberg adjusted *p*-values are compared by each method; significant results are marked with an asterisk (*).

**Table 2:** Significant results of logistic regression.

Feature parent feature gene method	Estimate	SE	z-value	*p*-value	adjusted *p*-value^a^
Co-occurrence					
Contrast					
TP53					
Phantom	9.66E−07	6.30E−07	1.535	1.25E−01	1
ComBat (Global)	1.879	0.498	3.771	1.63E−04	0.0081
ComBat (Reference 1)	1.839	0.490	3.754	1.74E−04	0.0087
ComBat (Reference 2)	1.869	0.501	3.733	1.89E−04	0.0095
Limma	1.855	0.494	3.751	1.76E−04	0.0088
Dissimilarity					
TP53					
Phantom	4.65E−06	4.45E−06	1.045	2.96E−01	1
ComBat (Global)	0.413	0.120	3.445	5.71E−04	0.0286
ComBat (Reference 1)	0.402	0.117	3.424	6.18E−04	0.0309
ComBat (Reference 2)	0.415	0.122	3.412	6.45E−04	0.0322
Limma	0.408	0.119	3.431	6.01E−04	0.0300
Intensity size zone					
Size-zone variability					
TP53					
Phantom	0.001	0.001	1.535	1.25E−01	1
ComBat (Global)	0.536	0.143	3.753	1.75E−04	0.0087
ComBat (Reference 1)	0.522	0.140	3.730	1.92E−04	0.0096
ComBat (Reference 2)	0.544	0.146	3.721	1.99E−04	0.0099
Limma	0.532	0.142	3.751	1.76E−04	0.0088
Neighborhood intensity difference					
Coarseness					
TP53					
Phantom	− 21.324	5.810	− 3.670	2.42E−04	0.0121
ComBat (Global)	− 28.487	7.783	− 3.660	2.52E−04	0.0126
ComBat (Reference 1)	− 30.392	8.336	− 3.646	2.67E−04	0.0133
ComBat (Reference 2)	− 21.695	5.860	− 3.702	2.14E−04	0.0107
Limma	− 25.074	7.428	− 3.376	7.37E−04	0.0368
Strength					
TP53					
Phantom	− 0.022	0.006	− 3.721	1.98E−04	0.0099
ComBat (Global)	− 0.260	0.076	− 3.431	6.02E−04	0.0301
ComBat (Reference 1)	− 0.275	0.081	− 3.412	6.46E−04	0.0323
ComBat (Reference 2)	− 0.198	0.058	− 3.441	5.80E−04	0.0290
Limma	− 0.224	0.072	− 3.100	1.94E−03	0.0968
Neighboring gray-level dependence					
Number nonuniformity					
TP53					
Phantom	4.02E−04	4.06E−04	0.989	3.22E−01	1
ComBat (Global)	0.401	0.122	3.299	9.72E−04	0.0486
ComBat_Ref1	0.390	0.119	3.277	1.05E−03	0.0524
ComBat_Ref2	0.404	0.124	3.266	1.09E−03	0.0545
Limma	0.394	0.121	3.271	1.07E−03	0.0536
Normalized co-occurrence					
Entropy					
TP53					
Phantom	0.856	0.217	3.948	7.89E−05	0.0039
ComBat (Global)	0.127	0.031	4.131	3.61E−05	0.0018
ComBat (Reference 1)	0.124	0.030	4.113	3.91E−05	0.0020
ComBat (Reference 2)	0.128	0.031	4.113	3.90E−05	0.0020
Limma	0.128	0.031	4.170	3.04E−05	0.0015
SUV statistics					
Entropy					
TP53					
Phantom	2.319	0.640	3.625	2.89E−04	0.0145
ComBat (Global)	0.532	0.141	3.763	1.68E−04	0.0084
ComBat (Reference 1)	0.528	0.139	3.803	1.43E−04	0.0071
ComBat (Reference 2)	0.571	0.150	3.811	1.38E−04	0.0069
Limma	0.549	0.143	3.844	1.21E−04	0.0060
Mean SUV					
IRS2					
Phantom	0.673	0.171	3.929	8.53E−05	0.0043
ComBat (Global)	0.624	0.188	3.311	9.29E−04	0.0465
ComBat (Reference 1)	0.696	0.209	3.323	8.91E−04	0.0445
ComBat (Reference 2)	0.360	0.138	2.618	8.84E−03	0.4419
Limma	0.644	0.209	3.083	2.05E−03	0.1026
SUV SD					
TP53					
Phantom	2.86E−04	0.005	0.059	9.53E−01	1
ComBat (Global)	2.264	0.684	3.307	9.41E−04	0.0471
ComBat (Reference 1)	2.498	0.725	3.448	5.65E−04	0.0283
ComBat (Reference 2)	1.881	0.577	3.262	1.11E−03	0.0554
Limma	1.930	0.639	3.019	2.54E−03	0.1270
Voxel alignment					
Intensity variability					
TP53					
Phantom	0.001	0.001	0.753	4.51E−01	1
ComBat (Global)	0.944	0.273	3.462	5.36E−04	0.0268
ComBat (Reference 1)	0.922	0.268	3.443	5.75E−04	0.0287
ComBat (Reference 2)	0.945	0.276	3.430	6.04E−04	0.0302
Limma	0.929	0.271	3.433	5.97E−04	0.0299

^a^*p*-values are adjusted using the Benjamini–Hochberg method.

## Discussion

In this study, the metabolic texture features of FDG PET/CT images from different instruments were unified by three correction methods: phantom correction, ComBat, and Limma. The uncorrected data demonstrated high batch effects compared to the corrected data. Although kBET rejection rate and silhouette score were lower in the phantom-corrected data than in the uncorrected data, the PCA plot showed similar variance. The ComBat and Limma methods provided correction with low batch effects, and there was no significant difference between the two methods. In ComBat- and Limma-corrected data, more texture features exhibited significant associations with the TP53 mutation than in the phantom-corrected data.

In practice, different PET/CT instruments from various vendors are utilized at different medical institutions and even within the same institute. Moreover, preparation of patients or image acquisition protocols differs according to the institute and region. There have been controversies whether there are significant differences in conventional image parameters, such as SUVmax, according to the instruments used^18,19^. Nevertheless, it is commonly acceptable to use conventional image parameters from different devices in clinical fields or studies owing to the overall similarity of examination procedures and image acquisition mechanisms. However, the instrument issue remains highly critical in the radiomics field. Previous studies suggested that radiomic features are sensitive to different acquisition methods and reconstruction parameters^20,21^. The heterogeneity of radiomic parameters between different instruments hinders employment of data from various scanners in clinical studies^22^. Therefore, pre-processing of radiomic data to remove batch effects is the most important step before conducting further analysis.

A phantom correction method is a conventional and basic approach to reduce the difference between each instrument. A cylinder phantom suggested by the National Electrical Manufacturers Association is widely used in performance test of PET/CT scanners^23,24^. As a measurement tool of the count rate or uniformity, it is used in quality control of scanners. Phantom correction has the advantage of identifying actual differences of image parameters from the measurement of a real phantom. However, it has two disadvantages. First, it is difficult in practice to acquire a reproducible correction coefficient between phantom images for various PET/CT scanners. Even in the same instrument, the measurement results may be changed each time according to different technicians or researchers. In addition, ambiguity exists in terms of which instrument is set as a reference if there are more than three scanners. Second, an appropriately heterogeneous phantom cannot be used. In real tumor tissue, there are few cases with high homogeneity along the whole tumor tissue. Nonetheless, a commonly used phantom can only provide a highly homogenic image owing to its simple structure. In the present study, the phantom-corrected data showed high variance in the PCA plot despite the low kBET rejection rate and silhouette score. Thus, phantom correction was deemed inferior to correction with ComBat and Limma methods with respect to practical limitations and clinical usefulness. It underscores the importance of integrating statistical methods like ComBat and Limma alongside physical correction techniques to achieve comprehensive harmonization for further application or research.

There were more metabolic texture features that showed a significant association with the TP53 mutation in the ComBat-corrected data and Limma-corrected data than in the phantom-corrected data. This finding suggests that correction with ComBat or Limma methods can more sensitively detect a notable association in radiogenomics studies. The inherent batch effects in the uncorrected or even phantom-corrected data seem to have obscured true biological associations. Most texture parameters that showed a significant association in ComBat-corrected data overlapped with those in Limma-corrected data. Thus, ComBat and Limma methods is deemed to be comparable for utilization in downstream analysis. Nevertheless, it is difficult to absolutely define and verify if the result is false-negative or false-positive. Nygaard et al. suggested that methods removing batch effects, such as ComBat harmonization, may exaggerate the significance of downstream analyses^25^. With this consideration, it cannot be ignored that ComBat-corrected data may demonstrate false-positive texture parameters associated with the TP53 mutation (co-occurrence contrast, co-occurrence dissimilarity, etc.). Therefore, care should be taken in interpreting results and presuming biological meanings in radiogenomics research. Also, further analyses based on large-scale image data from a single scanner are warranted to evaluate the possibility of false positives. It is noteworthy that distinct patterns were observed post-correction with all methods, with certain texture features such as coarseness and entropy being consistently associated with TP53 mutation. This highlights the possibility that TP53 mutation may influence tumor heterogeneity, which is then reflected in radiomic features. By understanding these associations, insights can be gained into the image characteristics linked to genetic changes in lung cancer, thereby bridging the gap between tumor biology and its observable phenotype. Nevertheless, given the limited number of features displaying associations and the absence of external validation, it is challenging to conclusively determine a distinct trend between radiomic features and genetic profiles. Further research is warranted in this regard.

In the present study, both the Limma and ComBat methods were applied for harmonization. As an analytic tool, Limma was originally developed for genomic data such as RNA-sequencing or microarrays^14^. ComBat employs a Bayesian framework, assuming that batch effects can be modeled as shifts in location and scale. It estimates batch-specific parameters and adjust the data accordingly. On the other hand, Limma assumes under the assumption that batch effects can be represented as linear additive factors. It fits a linear model to the data, includes batch as a covariate, and then removes the estimated batch effect. As radiomics analysis provides diverse features from medical images, radiomics data and genomics data are common in high-dimensional data across individuals. The ComBat method has been widely used for harmonization of radiomic data^26^. There are a few studies applying the Limma method for radiomic data. A previous study utilized the Limma method for selection of differentially expressed radiomic features not for harmonization^27^. Another study found that radiomics models harmonized with Combat and Limma data were not different for predicting neoadjuvant chemotherapy efficacy in breast cancer^28^. It is supported by the present study that demonstrated good accordance of low batch effects and genomic data associations between results from ComBat and Limma methods. It is noteworthy that this study is the first study to investigate value of Limma method for harmonization of radiomic data from FDG PET/CT images in terms of association with genomic data. While batch effect correction methods such as ComBat and Limma are valuable tools for mitigating the impact of technical variation, they introduce the potential risk of over- or under-correction in radiomics data analysis. One limitation of ComBat and Limma is their underlying assumption of additive batch effects, which may not fully capture the complexity of batch effect structures inherent to radiomics data. Additionally, the challenge arises from the difficulty in accurately assessing the precise nature and extent of these batch effects, given the multifaceted and high-dimensional nature of radiomics data. Over-correction can lead to the unintended removal of genuine biological variation, diminishing the biological insights that can be gleaned from the data. This concern is further exacerbated by the intricate and multifaceted nature of biological variation in radiomics data, which often involves complex interactions between features and may not be fully disentangled by these methods. Consequently, researchers must exercise caution in interpreting results, as the challenge of striking the right balance between batch effect removal and preservation of biologically relevant signal remains an ongoing consideration in radiomics data analysis.

For a reliable and reproducible radiomic model, methodologic basis, such as autosegmentation, data processing, and correction is essential^25,26,29^. In addition, given the current trend creating novel and multidisciplinary approaches based on different types of clinical information and other characteristics, addressing the data heterogeneity is of utmost importance^30^. Especially in the realm of radiogenomic research, the criticality of addressing batch effects cannot be understated. Previous literature may have marginalized this issue, risking the obfuscation of genuine biological associations indispensable for precise clinical interpretation. This study elucidates respective efficacies of batch correction methods, highlighting the importance of data harmonization in radiomic studies. This focus on rigorous batch effect correction becomes even more crucial in the context of multi-center investigations where data variability is an inherent challenge. By ensuring that radiomic parameters robustly represent true tumor biology, it is possible to obtain more precise, reproducible, and clinically relevant findings. Therefore, the present study underscores the necessity for meticulous radiomic data preprocessing in clinical field and oncology research. Furthermore, it is anticipated to enhance the reliability of previous investigations pertaining to the prognostic value of radiomic features, thereby augmenting their potential applicability^31,32^.

This study had several limitations. First, there was no evaluation for reproducibility of the phantom correction method. We produced a reference ratio between two instruments from a single experiment. However, it is reasonably hypothesized that a reference ratio would not differ significantly if a cylinder phantom is filled with an FDG solution with the most homogeneous concentration. Furthermore, even if there is significant non-reproducibility in a phantom correction method, it would support our discussion to suggest the practical superiority of a harmonization method. Second, only two different instruments were used in this study. In multi-center trials, FDG PET/CT images from three or more scanners may be enrolled. A further study with more scanners would more strongly support the substitutability of the harmonization method. Third, several patients were excluded due to different therapeutic history or quality control. Generally, excluding patients is considered a potential source of selection bias. However, incorporating genomic data from treated tissues may not be accurate, as they don’t reflect inherent properties of untreated tumors. Using data without quality control can compromise study results, and including atypical lung cancers might limit the study’s broader applicability. Thus, it is contended that our exclusions in fact enhance the study’s reliability and generalizability. Fourth, despite of harmonization, there is an inherent limitation of heterogeneity stemming from target segmentation, and feature extraction algorithms^31^. While uniformity can be partially achieved in same methods of target segmentation or feature extraction software, aligning all conditions equally is a significant challenge. Finally, this study focused on the association between the texture parameters and the genetic characteristics of lung cancer. Although genomic data have been widely used in recent oncology fields, clinical outcomes, such as disease-free survival and overall survival, remain the most important factors in clinical practice and research. Further investigation is warranted to focus on the correlation between the corrected texture parameters and clinical outcomes in various cancer types beyond lung cancer.

In conclusion, ComBat- and Limma-corrected data showed fewer batch effects than phantom-corrected and uncorrected data. ComBat and Limma correction reduced batch effects with no significant difference between the two methods. In ComBat and Limma-corrected data, more texture features demonstrated a significant association with the TP53 mutation than those in phantom-corrected data. These findings suggest that correction with ComBat or Limma methods can be more effective or equally as effective at reducing batch effects than correction with the phantom method. Despite the possibility of false-positive findings, ComBat-corrected data or Limma-corrected data may be acceptable for use in further analyses considering their practical availability and results with comprehensible association with genetic characteristics.

## Data Availability

The data generated in this study are available upon request from the corresponding author.
